# Dominative role of MMP-14 over MMP-15 in human urinary bladder carcinoma on the basis of its enhanced specific activity

**DOI:** 10.1097/MD.0000000000019224

**Published:** 2020-02-14

**Authors:** Jacek Kudelski, Grzegorz Młynarczyk, Barbara Darewicz, Marta Bruczko-Goralewska, Lech Romanowicz

**Affiliations:** a Department of Medical Biochemistry; b Department of Urology, Medical University of Białystok, Poland.

**Keywords:** MMP-14, MMP-15, transmembrane matrix metalloproteinases, urinary bladder carcinoma

## Abstract

**Background:**

Human urinary bladder cancer is one of the most common cancers worldwide with the mortality rate of approximately 165,000 people annually. The modulation of extracellular matrix is a crucial event in the metastatic spread, among others in angiogenesis. It is initiated and prolonged by the cascade of matrix metalloproteinases. MMP-14 and MMP-15 are associated with a high degree of malignancy, aggressiveness, and survival prognosis by the activation of other matrix metalloproteinases (MMPs). This study was aimed at evaluating the expression and the activity of selected transmembrane metalloproteinases at different stages of human urinary bladder cancer.

**Methods:**

Western blot and enzyme linked immunosorbent assay (ELISA) method were used to evaluate the expression and content of MMPs and TIMP-1. The activity of studied enzymes was determined with fluorometric method.

**Results:**

Both transmembrane metalloproteinases are found in healthy or cancerous tissue in high molecular complexes of human urinary bladder. MMP-14 dominates over MMP-15, particularly in high-grade urinary bladder cancer. Their contents significantly change with the grade of bladder tumor. The amount of MMP-14 increases with increasing grade of tumor. MMP-15 content decreases in high-grade bladder cancer. With increasing grade of urinary bladder cancer their actual activity (per kg of total protein content) is varying in different ways. In all examined tissues, the specific activity of MMP-15 (per kg of the enzyme content) is much higher in comparison to MMP-14. Human urinary bladder cancer contains higher TIMP-1 amounts than control tissue but with the decrease with an increase in tumor grade.

**Conclusion:**

Comparison of investigated enzymes’ activity and the inhibitor content suggests it opposite effects, higher suppression of MMP-14 than MMP-15 activity in low-grade bladder cancer and reverse TIMP-1 action in high-grade cancer. The MMP-14 activity determination in urinary bladder cancer tissue may be used as a predictor of a risk of metastasis.

## 1 Introduction

According to current knowledge, there are 28 matrix metalloproteinases, whereas 23 occur in the human organism. Their main function consists in degrading extracellular matrix components. Their proteolytic activity is regulated by transcription factors, endogenous inhibitors, and proteases. According to recent reports, matrix metalloproteinases (MMPs) modulate multiple tumor-supporting cellular processes such as cell proliferation, angiogenesis, and apoptosis.^[1–6]^

The urinary bladder collects the urine produced by kidneys. The bladder itself includes 4 layers, which are important landmarks in determining how deeply the tumor has invaded and what the ultimate stage of the cancer is. The type of cancer is also described by its grade under microscope. Low-grade tumors have a much lower risk of progression than high-grade tumors. Early disease detection in individuals who are at high risk is very important.^[7–10]^

They are formed by 6 enzymes. Four of them are classified as transmembrane type I proteins (MMP-14, MMP-15, MMP-16, and MMP-24). These enzymes degrade: collagen types I, II, and III, aggrecan, elastin, fibronectin, gelatin, laminin, MMP-2, -13. Except for MMP-17, they all have a specific function of proMMP-2 activation.^[11–13]^ Their activity is regulated by tissue inhibitors of matrix metalloproteinases (TIMPs). All 4 TIMPs have the ability to bind 2 different places in membrane-type metalloproteinases molecule.^[2,11]^

MMP-14, also called membrane-type 1 matrix metalloproteinase (MT1-MMP), plays a very important role in the angiogenesis by degradation of collagen types I, II, and III. It promotes cellular invasion in cancerogenic processes, metastasis, angiogenesis, wound healing, atherosclerosis, and rheumatoid arthritis.^[14,15]^ MMP-14 enhances the local invasion of the tumor and the formation of metastases via the activation of pro-MMP-2. An enhanced expression of MT1-MMP seemed to be associated with a high degree of malignancy, aggressiveness, and survival prognosis.^[12–16]^

It was shown that MMP-15 (MT2-MMP) may promote cell invasion into basement membrane matrices^[17–19]^ by MMP-2 activation. In a few research studies, the expression of MT2-MMP was markedly higher in tumors with a higher degree of aggressiveness and malignancy.^[15]^

## 2 Aim of the study

The content and activity of MMPs in urinary bladder cancer was the object of various research studies whose outcomes often differed as a result of variations in methods and tissue material. Dominant outcomes were determined in blood and urine of patients with cancer. It seems that the outcomes do not fully correspond to the real participation of MMPs in the tissue changed by carcinogenesis. Based on abovementioned observation, the expression, content, and activity of MMP-14 and MMP-15 in the cancerous tissue in comparison with control tissue were evaluated.

## 3 Materials and methods

The study protocol was approved by the Bioethical Committee of the Medical University of Bialystok.

### 3.1 Tissue material

The samples to be investigated were taken in the course of surgeries performed at the Department of Urology, Medical University of Bialystok, from urothelial cancer in 2 phases of morphological malignancy. The participants of the study were 20 patients after radical cystectomy or transurethral resection of bladder tumor in whom the urothelial cancer was histopathologically diagnosed. The age range was 47 to 91, the average was 70.3.

The classical, open method was used to excise a urinary bladder, then a tissue sample was collected from the macroscopically visible tumor. The study was conducted on 10 patients diagnosed with a low-grade cancer and 10 patients diagnosed with a high-grade cancer. The control tissue was taken from the side opposite to the tumor, following a radical open cystectomy procedure. During the transurethral resection of the bladder tumor, it was not possible to collect the healthy tissue. The following abbreviations will be used with reference to the material: LG – low-grade cancer, HG – high-grade cancer, and Control – control tissue.

### 3.2 MT-MMP's and TIMP-1 content

The MMP-14 content in the evaluated materials was determined with the quantitative assay, Human (MMP-14) enzyme linked immunosorbent assay (ELISA) Kit and the MMP-15 content with Human (MMP-15) ELISA Kit (both provided by Shanghai Sunred Biological Technology, China) and Human TIMP-1 (Tissue Inhibitors of Metalloproteinase 1) ELISA Kit (Elabscience Biotechnology, China) according to instructions given by the manufacturers.

*MT-MMP's and TIMP-1 Western blot* analysis was made with the use of respective monoclonal antibody.^[20]^

*MT-MMP's activity* was measured with fluorogenic substrate.^[21]^ The MMP activity was expressed in katals per kg of protein.

*Protein determination* was evaluated with the use of the Bradford^[22]^ protein assay.

### 3.3 Statistical analysis

The performed calculation gave mean values of 10 assays ± standard deviations (SD). The matrix metalloproteinases content was expressed in nmol/g of fresh tissue. Their activity was given in microkat/kg of protein. The Student's *t* test was used for the statistical analysis, with the significance at the level of *P* < .05.

## 4 Results

### 4.1 MMP-14, MMP-15, and TIMP-1 content

Both transmembrane metalloproteinases were present in urinary bladder and in bladder cancer and were given in milligrams per kg of protein (Table 1). MMP-14 present in the control tissue extract amounted to 7.45 mg/kg of protein. Low-grade and high-grade urinary bladder cancers demonstrated significantly higher amounts of that enzyme, namely almost 35% more of the enzyme in low-grade cancer and an especially high value, about more than 10 times of the enzyme, in high-grade cancer compared to the control tissue (Table 1).

**Table 1:**
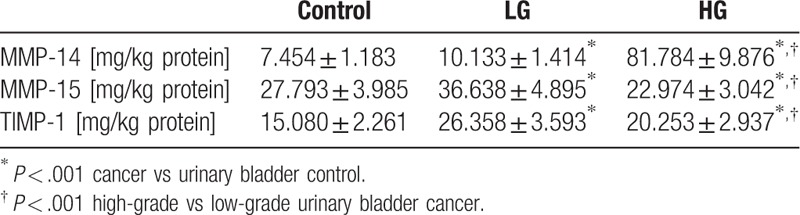
Total content of MMP-14, MMP-15, and TIMP-1 in control human urinary bladder and its cancers.

The MMP-15 content in a normal urinary bladder wall was more than 3 times higher compared with the MMP-14 content in the same tissue. Low-grade cancer was characterized by a higher amount of MMP-15 while its content in high-grade cancer was 4 mg/kg lower than in the control tissue (Table 1).

The lowest content of TIMP-1 was found in control urinary bladder (Table 1). The highest content of the inhibitor was determined in low-grade cancer tissue. The value was almost 75% higher than in control tissue. The amount of TIMP-1 significantly decreased in high-grade cancer tissue but still it was higher in comparison to control (Table 1).

### 4.2 Western blot analysis of investigated transmembrane metalloproteinases and TIMP-1

**Figure 1:**
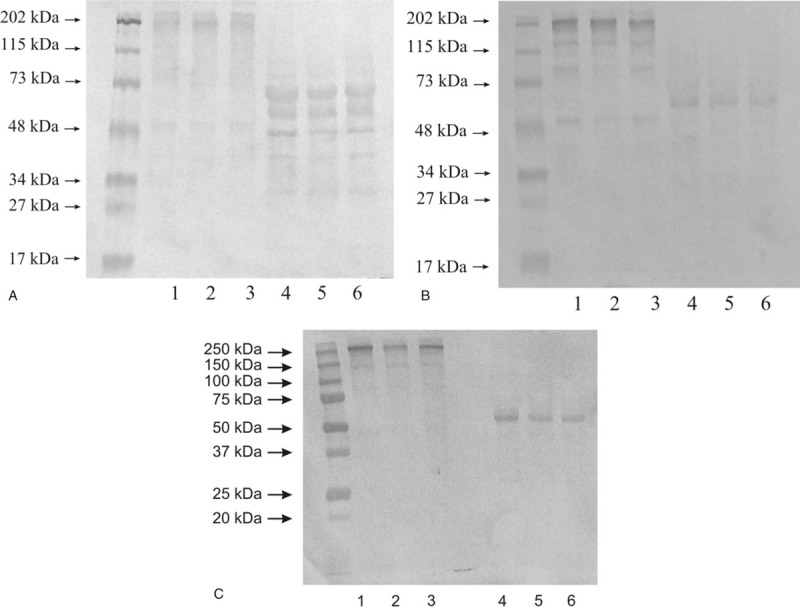
Western Immunoblot of MMP-14 (A), MMP-15 (B), and TIMP-1 (C) in control tissue and low-grade (LG) and high-grade (HG) urinary bladder cancer. Samples contained 20 μg of protein was applied on each lane. Non-reducing conditions: Lane 1 – control urinary bladder, 2 – low-grade bladder cancer, 3 – high-grade bladder cancer. Reducing conditions: Lane 4 – control urinary bladder, 5 – low-grade bladder cancer, 6 – high-grade bladder cancer.

The electrophoresis for western blot analysis was conducted in non-reducing and reducing conditions with the same protein amount in each sample. The representative results are presented in Figure 1.

#### 4.2.1 Expression of MMP-14 in human urinary bladder

The findings of western blot analysis of MMP-14 expression in a normal urinary bladder and in tissues changed by carcinogenic processes were presented in Figure 1A. We applied 20 μg of protein on lane 1 to 3, and 20 μg of protein on lane 4 to 6 of the same samples. Normal urinary bladder demonstrated very thin and very little visible bands with a molecular mass of 202, 48 kDa (lane 1). The low-grade urinary bladder cancer tissue showed similar results as the control tissue (lane 2). The high-grade urinary bladder cancer tissue demonstrated the same outcomes as the control and low-grade cancer tissues (lane 3). A reduction of disulfide linkages resulted in the visualization of thicker bands. All 3 kinds of samples demonstrated 3 bands with a molecular mass of 65, 55, and 48 kDa (lane 4–6) shown in Figure 1A.

#### 4.2.2 Expression of MMP-15 in human urinary bladder

The findings of western blot analysis of MMP-15 expression in a normal urinary bladder and in tissues changed by carcinogenic processes were presented in Figure 1B. We applied 20 μg of protein on lane 1 to 3, and 20 μg of protein on lane 4 to 6 of the same samples. Normal urinary bladder demonstrated at least 4 bands with a molecular mass of 202, 120, 80, and 50 kDa (lane 1). The tissues of low-grade urinary bladder cancer (lane 2) and high-grade urinary bladder cancer (lane 3) demonstrated outcomes similar to the control tissue. The condition with a reduction of disulfide linkages resulted in a reduction of the number of visible bands. The anti-MMP-15 antibody reacted with proteins with the molecular mass of about 66 kDa (lane 4). Low-grade and high-grade bladder cancer tissues demonstrated similar results (lane 5 and 6) presented in Figure 1B.

#### 4.2.3 Expression of TIMP-1 in human urinary bladder

As could be seen in Figure 1C, the most intensive band on Western blot analysis of TIMP-1 expression without disulfide bond reduction in healthy urinary bladder and its cancer at different stadium was around 250 kDa with similar intensity for all investigated tissues (lane 1–3). Much weaker bands were with molecular mass of 150 kDa. Under the action of reducing agent the band with the highest molecular mass disappeared for all tissue samples. Instead of that, a band with around 60 kDa was visible for control tissue (lane 4), low-grade bladder cancer (lane 5), and high-grade cancer (lane 6). There was no visible band representing free form of TIMP-1 for all examined tissues independently with or without reducing agent (Fig. 1C).

### 4.3 Actual activity of MMP-14 and MMP-15

**Figure 2:**
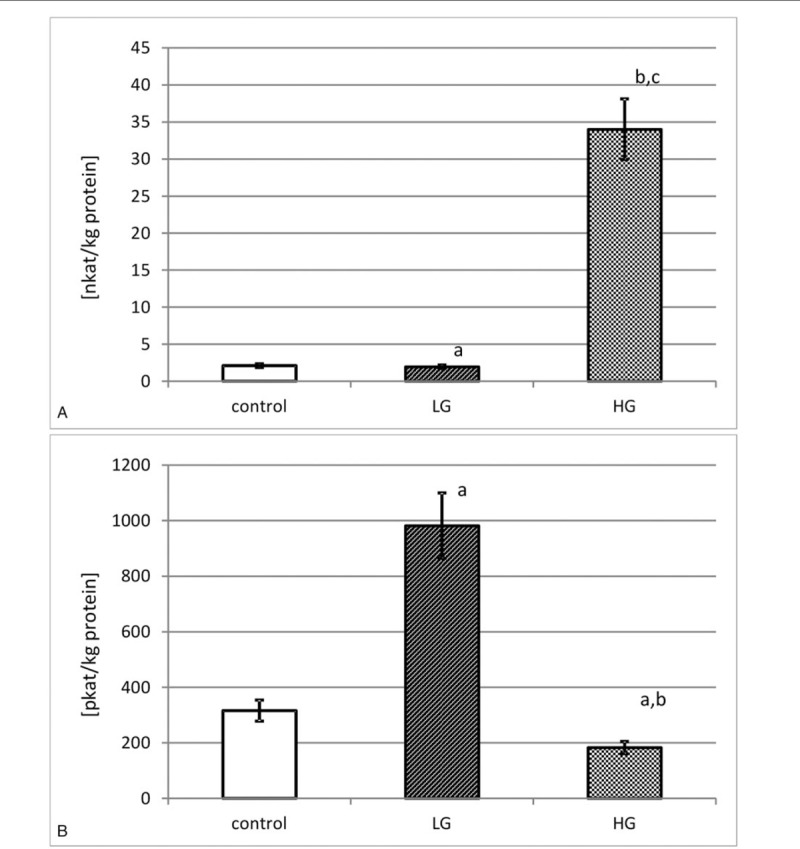
Actual activity of MMP-14 (A) and MMP-15 (B) in control tissue and low-grade (LG) and high-grade (HG) urinary bladder cancer. MMP-14: a – *P* < .05 low-grade cancer vs urinary bladder control. b – *P* < .001 high-grade cancer vs urinary bladder control. c – *P* < .001 high-grade vs low-grade urinary bladder cancer. MMP-15: a – *P* < .001 cancer vs urinary bladder control. b – *P* < .001 high-grade vs low-grade urinary bladder cancer.

The actual activity of collagenases was measured by means of the fluorimetric method with oligopeptide acting as a substrate. Each enzyme was isolated on a microplate pre-coated with an antibody specific for assayed collagenase, the same which was used for Western Immunoblot. The actual activity was given in katals per kg of total protein content in tissue extract (Fig. 2).

#### 4.3.1 Actual activity of MMP-14

As can be seen from Figure 2A, the actual activity of MMP-14 was almost 2.1 nkat/kg of protein in normal human urinary bladder. The tissues of low-grade urinary bladder cancer were characterized by a little reduction of the actual activity of MMP-14. The measured activity rose with the increase in grade of urinary bladder cancer. There were significant differences between both grades of cancer and the control tissue (Fig. 2A).

#### 4.3.2 Actual activity of MMP-15

According to the results presented in Figure 2B, the actual activity of MMP-15 was about 316 pkat/kg of total protein in normal human urinary bladder. The actual activity of MMP-15 found in low-grade urinary bladder cancer was more than 3 times higher. An increase in the cancer grade brought about a decrease in actual activity of MMP-15 by nearly 2 times. There were significant differences in actual activity of MMP-15 between both grades of cancer and the control tissue (Fig. 2B).

### 4.4 Specific activity of MMP-14 and MMP-15

**Figure 3:**
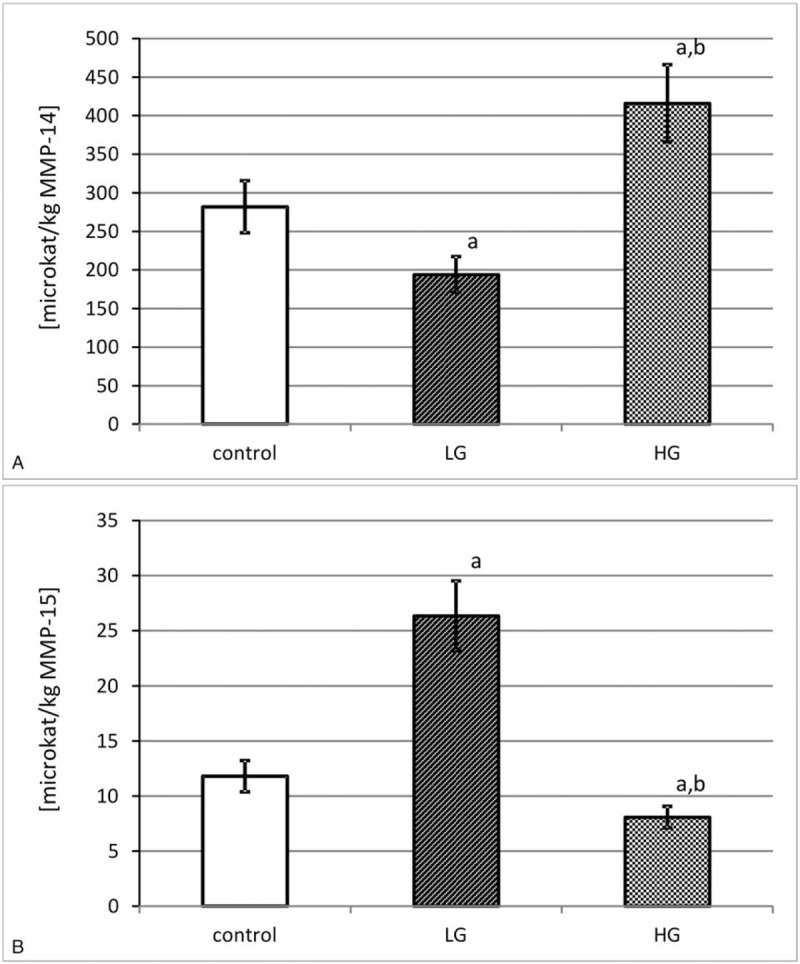
Specific activity of MMP-14 (A) and MMP-15 (B) in control tissue and low-grade (LG) and high-grade (HG) urinary bladder cancer. a – *P* < .001 cancer vs urinary bladder control. b – *P* < .001 high-grade vs low-grade urinary bladder cancer.

The calculation of specific activity for each transmembrane MMP was based on the results of actual activity of the enzyme and its content measured separately for each patient by the ELISA technique. The mean value of specific activity was calculated and was expressed in katals per kg of respective enzymatic protein content in the tissue extract (Fig. 3).

#### 4.4.1 Specific activity of MMP-14

As can be seen from Figure 3A, the highest specific activity of MMP-14 was found in high-grade urinary bladder cancer. The calculated specific activity of MMP-14 was 2 times lower in low-grade urinary bladder cancer. There were significant differences between both grades of cancer and the control tissue (Fig. 3A).

#### 4.4.2 Specific activity of MMP-15

The outcomes of MT-MMP-2 specific activity were presented in Figure 3B in microkatals per kg of enzyme. The control tissue showed a middle value of that activity. The low-grade urinary bladder cancer was distinguished by a significant increase in specific activity of MMP-15. The high-grade of cancer decreased that activity by nearly 4 times (Fig. 3B).

## 5 Discussion

Urinary bladder plays an important role in the human organism. Any pathological process occurring in urinary bladder and ending with a cancer may lead to structural and functional damage of this vital organ. As a result, painful urination or blood in urine may occur in the course of other diseases. The percentage of deaths as a result of urinary bladder cancer is very high. Malignant tumors of the bladder cause in men about 5% and in women 2% of all cancer deaths. The risk of dying from this cancer increases with age from the sixth decade of life and reaches its highest values after the age of 80. In Poland, the incidence of bladder cancer was lower than the average for European Union countries in both sexes. Mortality from bladder cancers in Poland is higher among men than the average for European Union countries.^[23]^ Therefore, it is a subject of interest for many investigators.

Besides the structural function, the extracellular matrix of the bladder has a primary role in organizing the tissue architecture. Furthermore, structural proteins of the wall are essential in maintaining the integrity of the impermeable bladder surface.^[24]^ As a dynamic structure, it plays a key role in cell proliferation, differentiation and migration, tissue repair and angiogenesis as well as in modulation of tumor cell invasion and metastatic processes.^[25–27]^

Collagen is the main extracellular matrix protein of a healthy urinary bladder. Its large amount is associated with its extensibility function incident to the filling of the bladder lumen.^[9]^ The degree of collagen degradation is contingent on the presence and the activity of matrix metalloproteinases. In urinary bladder cancer, the content of studied metalloproteinases varied significantly and was related to the cancer progression stage.

In particular, the MMP-15, also referred as MT2-MMP, content found in healthy bladder was more than 3 and a half times higher as compared to MMP-14 (MT1-MMP). It suggested that MMP-15 was more crucial for the reconstruction of extracellular matrix in the urinary bladder. The MMP-15 amount was over 3 times higher than MMP-14 also in low-grade cancer tissues. But this relationship changed with the grade of tumor. The content of MMP-14 in the high-grade urinary bladder tissue was nearly 4 times higher in comparison to MMP-15. The MT1-MMP content was over 8 times higher in high-grade tumors than in low-grade tumors. The high-grade urinary bladder cancer contained MT2-MMP at a lower level than at the low-grade stage, being very similar to the control tissue. A much higher MMP-14 content in the higher stage of malignancy as compared to MMP-15 and a completely different relationship at the low-grade stage were worth noting. These results showed that the synthesis and the secretion of metalloproteinases out of cells were not inhibited.^[6,11,12,14]^

The expression of both transmembrane metalloproteinases determined by Western Immunoblot showed some differences for all investigated tissues except MMP-14 and MMP-15 for high molecular masses 202 kDa in a condition without the reduction of disulfide linkages. Band with a molecular mass of about 55 kDa for MMP-14^[28]^ and of about 66 kDa for MMP-15^[29]^ visible after disulfide bond reduction might constitute a free active form of the respective enzyme. Bands having a higher molecular mass revealed the presence of investigated metalloproteinases in complexes with other extracellular matrix proteins including TIMPs, or even the dimer formation. The extracellular matrix is a specific part of the tissue where various proteins interacted with one another, which in many cases did not influence the enzyme activity.^[9,10]^

Based on the measurement of actual activity, expressed in katals per kilogram of total protein content in the tissue extract, it was possible to compare the investigated transmembrane MMPs activities. There were differences in the actual activity of both MMPs in normal bladder tissue. It was much higher of MMP-14 compared to MMP-15 as it was expressed in nanokatals while for MMP-15 it was stated in pikokatals per kilogram of the total protein content. In view of the permanent process of remodeling of extracellular matrix with the participation of its metalloproteinases, it appeared that MMP-14 was involved to a great extent in maintaining the homeostasis of extracellular matrix in normal urinary bladder. In both grades of urinary bladder cancer, the actual activity of membrane-type 1-MMP was higher than of MT2-MMP. It might be regarded as further evidence that MMP-14 took a very important part in the extracellular matrix reconstruction process.^[5,6,12,13,15]^

The highest increase in TIMP-1 content was observed in low-grade urinary bladder cancer. Despite that, MMP-15 presented the highest activity at that stage of the cancer. It suggested that most of the inhibitors were bound to hemopexin domain that did not change the enzyme activity.^[2,11]^ At the same time, it was found that MMP-14 activity significantly decreased in low-grade cancer. Taking together with high amount of the inhibitor it seemed that TIMP-1 was bound to catalytic domain of the enzyme which involved in the inhibition of MMP-14 activity.^[2,11]^ The opposite effects were observed in high-grade urinary bladder cancer. The increase in MMP-14 activity with simultaneous decrease in TIMP-1 content suggested the release of the metalloproteinase from the influence of the inhibitor. The inverse changes were detected for MMP-15 in high-grade cancer. The reduction of the enzyme activity might be evoked by the higher content of the inhibitor.

Other studies showed that MMP-14 enhanced the local invasion and the formation of metastases via the activation of pro-MMP-2. The enhanced expression of MMP-14 appeared to be associated with the high degree of malignancy, the aggressiveness and the survival prognosis.^[12–15]^ Also the MMP-15 expression was distinctly higher in tumors with a higher degree of aggressiveness and malignancy. So far, very few papers regarding other transmembrane metalloproteinases and other MMPs have been published.^[15]^

As a result of evaluated specific activity, we were able to find what part of the enzyme appeared in an active form with an active center free of tissue inhibitors. The MMP-14 specific activity was higher with increasing histopathological grade of the tumor. The specific activity of MMP-15 decreased with increase of cancer histopathological grade. Membrane type-1 MMP proteins seemed to be more enzymatically active compared to MT2-MMP. Observed differences might indicate that they were specifically involved in a particular period of growth and a differentiation of the urinary bladder cancer. In addition, these results suggested that cancerous cells of the low-grade urinary bladder tumor might silence the activity of MMP-14. Such differences in the activity of both metalloproteinases suggested their contrary participation in extracellular matrix remodeling at different tumor development stages.^[23–25]^ Considering that the control material was taken from the same bladder, a carcinogenetic influence on the metabolism of the whole urinary bladder as an organ was possible. The initiation of collagen degradation in the extracellular matrix could be regarded as the most significant stage in the process of tumor growth. A better reflection of the role of transmembrane metalloproteinases in the tissue metabolism was achieved by the use of tissue material for the study instead of urine or blood serum of the same patient.

## 6 Conclusion

We found that the changes in the amount of membrane-type matrix metalloproteinases differed in healthy and cancerous human urinary bladder. Based on content outcomes only, it is not evident which enzyme was dominating in the urinary bladder cancer. The specific activity of investigated metalloproteinases was considerably higher for MMP-14 than for MMP-15, especially in the high-grade urinary bladder cancer. Comparison of investigated enzymes’ activity and the inhibitor content suggested it opposite effects, higher suppression of MMP-14 than MMP-15 activity in low-grade bladder cancer and reverse TIMP-1 action in high-grade cancer. Such findings pointed to differences in respect of the regulation of enzyme expression and activation. The MMP-14 activity determination in urinary bladder cancer tissue may be used as a predictor of a risk of metastasis.

## Author contributions

**Conceptualization:** Jacek Kudelski

**Data curation:** Grzegorz Młynarczyk

**Formal analysis:** Jacek Kudelski

**Investigation:** Grzegorz Młynarczyk

**Methodology:** Marta Bruczko-Goralewska

**Supervision:** Barbara Darewicz

**Validation:** Lech Romanowicz

**Writing – original draft:** Jacek Kudelski, Grzegorz Młynarczyk

**Writing – review & editing:** Barbara Darewicz, Lech Romanowicz
